# Whole-genome sequence of *Psychrobacter* sp. strain AH5, isolated from salted bluespot mullet (*Valamugil seheli*)

**DOI:** 10.1128/mra.00829-24

**Published:** 2024-11-22

**Authors:** Ahmed H. Al-Harbi

**Affiliations:** 1 Sustainability and Environment Sector, King Abdulaziz City for Science and Technology, Riyadh, Saudi Arabia; Montana State University, Bozeman, Montana, USA

**Keywords:** *Psychrobacter*, Bluespot mullet (*Valamugil seheli*), salted fish, whole-genome sequence

## Abstract

Herein is reported the whole-genome sequence of *Psychrobacter* sp. strain AH5 that was isolated from salted bluespot mullet (*Valamugil seheli*). The genome assembly comprises a total of 3,241,797 bp with a GC content of 43.73% and encodes 2,541 protein-coding genes.

## ANNOUNCEMENT

The genus *Psychrobacter* belongs to the family of *Moraxellaceae* within the class of *Gammaproteobacteria*. The members of this genus are Gram-negative coccobacilli, psychrotolerant, halotolerant, aerobic, and nonmotile bacteria, which are widely distributed in marine and terrestrial environments (1). *Psychrobacter* spp. have been isolated from several aquatic animal species, including fish (2, 3), lobsters (4), oysters (5), and shrimp (6, 7). Additionally, they have been recovered from a variety of salted and fermented seafood products (8–11).

*Psychrobacter* sp. strain AH5 was originally isolated from salted bluespot mullet (*Valamugil seheli*) muscle obtained from the fish market in Riyadh, Saudi Arabia (latitude: 24.145536 N; longitude: 47.311947 E) in 2017 as described previously (11). The strain AH5 was preserved at −80°C in tryptone soy broth containing 15% glycerol. The isolate was preliminarily identified as a member of the genus *Psychrobacter* by Vitek 2 Compact and 16S rDNA analysis (11).

For whole-genome sequencing, cryostocks of *Psychrobacter* sp. AH5 was revived and streaked onto tryptone soy agar plates, and a single colony was grown overnight at 30°C in 10 mL tryptone soy broth (Oxoid). Subsequently, the bacterial cells were harvested by centrifugation at 10,000 *g* for 10 min. Genomic DNA was extracted using a DNeasy blood and tissue kit (Qiagen) following the manufacturer’s instructions. DNA quantity, quality, and integrity were assessed using a NanoDrop spectrophotometer (Thermo Fisher Scientific, USA) and 1% agarose gel electrophoresis, respectively.

Genomic library was prepared from 1.5 µg of bacterial genomic DNA fragmented to an average size of ~470 bp using g-TUBEs devise (Covaris, Woburn, MA, USA) following the manufacturer’s instructions. Library was generated with NEBNext Ultra II DNA library preparation kit (New England Biolabs, Ipswich, MA, USA), visualized on a 2100 Bioanalyzer (Agilent Technologies, Inc., Santa Clara, CA, USA), and quantified by quantitative PCR (qPCR) with a KAPA library quantification kit (Thermo Fisher Scientific). Sequencing was performed using the NovaSeq 6000 (2 × 150 bp paired-end reads) (Illumina Inc., San Diego, CA, USA) platform. The sequence reads were trimmed for quality using Trimmomatic v.0.30 with a sliding window quality cutoff of Q30 (12) and assembled *de novo* using SPAdes v.3.15.3 (13) followed by polishing with Pilon v1.24 (14). Genome completeness was evaluated with Benchmarking Universal Single-Copy Orthologs (BUSCO) v5.3.2 (15). Finally, the assembly was annotated with the NCBI Prokaryotic Genome Annotation Pipeline (PGAP) v4.13 (16). Default parameters were used for all software unless otherwise specified.

**Fig 1:**
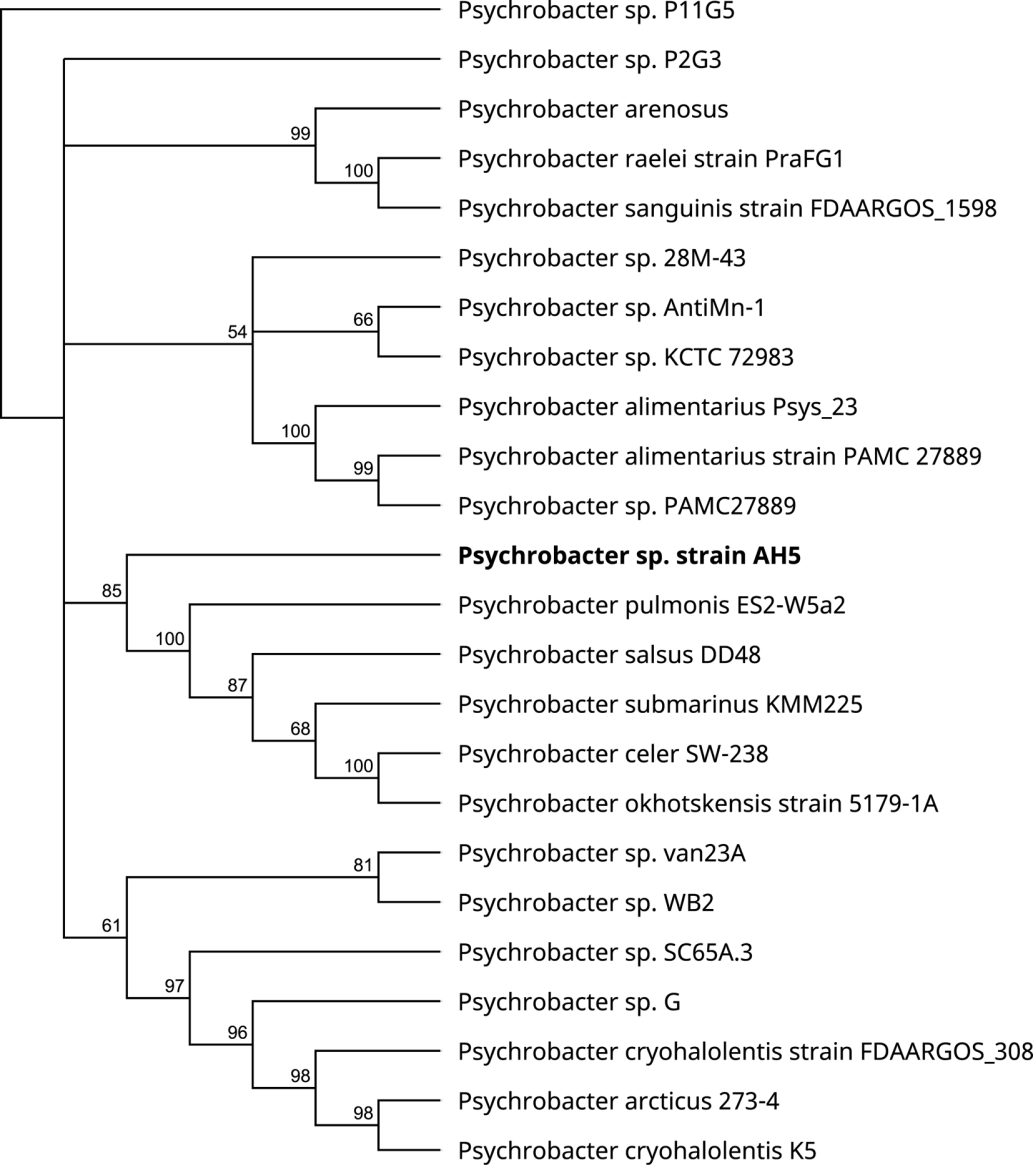
Neighbor-joining phylogenetic tree showing the placement of the strain *Psychrobacter* sp. AH5 within the genus. The nucleotide sequence was concatenated and subsequently aligned using Clustal Omega (v1.2.2, default settings) in Geneious Prime (Dotmatics, 2024.0.5). Bootstrap values are indicated at the nodes of the branches. The AH5 strain whose genome is reported in this announcement is highlighted in bold font.

The whole genome of *Psychrobacter* sp. AH5 contains a single linear chromosome of 2,967,972 bp, with a GC content of 43.53%. The main genome statistics and features of *Psychrobacter* sp. AH5 are summarized in Table 1. Phylogenetic tree based on the nucleotide sequence of *Psychrobacter* sp. strain AH5 and 23 publicly available references. The phylogenetic tree was constructed with the neighbor-joining method using the Clustal Omega program (v1.2.2, default settings) in Geneious Prime (Dotmatics, 2024.0.5) and subsequently aligned using MUSCLE (v3.8.425). A phylogenetic analysis revealed that the strain AH5 belongs to the genus *Psychrobacter*, forming an independent branch (Fig. 1). The average nucleotide identity (ANI) values generated using the OrthoANIu algorithm (v1.2) (17) showed that the strain AH5 genome sequence to the type strains of *Psychrobacter* species, with publicly available genome information, was below the 95% threshold. The closest reference genomes are *Psychrobacter salsus* DD48 (CAJHAW000000000) and *Psychrobacter submarinus* KMM225 (CAJHBQ000000000) with an ANI of 78.58% and 78.55%, respectively. Based on the phylogenetic tree and ANI values suggesting that the strain AH5 is a novel species of the genus *Psychrobacter*.

**TABLE 1:** Genome statistics and features of *Psychrobacter* sp. strain AH5

Parameter	Value
Genome project information	
BioProject accession no.	PRJNA830767
BioSample accession no.	SAMN27738345
Sequence Read Archive (SRA) accession no.	SRR18927018
GenBank assembly accession no.	GCA_040371085.1
Raw sequencing reads	
No. of reads	7,539,508
Total no. of bases	10,822,110,200
Avg genome coverage (x)	998.2651
Genome features	
Genome size (bp)	3,241,797
No. of contigs	7
GC content (%)	43.73
Total no. of genes	2,643
No. of coding sequences	2,581
No. of proteins	2,541
No. of pseudogenes	40
No. of rRNAs	10
No. of tRNAs	48
No. of noncoding RNAs	4
BUSCO results (%)	
Complete BUSCOs (%)	97.6
Single copy and complete BUSCOs (%)	97.6
Duplicated and complete BUSCOs (%)	0
Fragmented BUSCOs (%)	2.4
Missing BUSCOs (%	0

## Data Availability

The whole-genome sequence of *Psychrobacter* sp. strain AH5 has been deposited at DDBJ/ENA/GenBank under the accession number JAMBMT000000000. The BioProject, BioSample, assembly, and Sequence Read Archive (SRA) accession numbers are provided in Table 1. The version described in this paper is version JAMBMT010000000.
